# A comparative analysis of tip-bendable suction ureteral access sheath versus traditional sheath in retrograde intrarenal stone surgery: a systematic review and meta-analysis of comparative studies

**DOI:** 10.1097/JS9.0000000000002969

**Published:** 2025-07-23

**Authors:** Zhenlang Guo, Zehuai Wen, Jiwan Qiu, Guixing Tang, Franky Leung Chan, Zhaohui Wang, Zunguang Bai, Junwei He

**Affiliations:** a Department of Urology, The Second Affiliated Hospital of Guangzhou University of Chinese Medicine, Guangzhou, China; b Department of Statistics, The Second Affiliated Hospital of Guangzhou University of Chinese Medicine, Guangzhou, China; c Department of Urology, Meizhou Hospital of Traditional Chinese Medicine, Meizhou, China; d School of Biomedical Sciences, The Chinese University of Hong Kong, Shatin, Hong Kong, China

**Keywords:** meta-analysis, retrograde intrarenal stone surgery, suction, systematic review, tip-bendable, ureteral access sheath

## Abstract

**Objective:**

This systematic review and meta-analysis seeks to evaluate the efficacy and safety of tip-bendable suction ureteral access sheaths (TBS-UAS) compared to traditional ureteral access sheaths (UAS) in retrograde intrarenal stone surgery (RIRS).

**Methods:**

A thorough literature search was performed across multiple databases, including MEDLINE (via PubMed), Cochrane Library, and Embase (via Ovid) for studies published until January 2025. We included randomized controlled trials (RCTs) and observational studies that reported data on stone-free rates (SFRs), surgical duration, postoperative length of stay, and complication rates. Data were extracted and analyzed using random or fixed effects models to compute pooled relative risks (RRs) and mean differences (MDs), along with their corresponding 95% confidence intervals (CIs).

**Results:**

Eight studies involving a total of 1981 patients were included in the analysis. The TBS-UAS group exhibited a statistically significant enhancement in immediate SFR (RR = 1.57, 95% CI: 1.18–2.09) and SFR at 1 month postoperatively (RR = 1.24, 95% CI: 1.05–1.46) compared to the traditional UAS group. Additionally, the TBS-UAS was associated with a lower overall complication rate (RR = 0.41, 95% CI: 0.29–0.56) and a reduced incidence of fever (RR = 0.37, 95% CI: 0.25–0.53). No significant differences were found regarding surgical duration, postoperative length of stay, intraoperative bleeding, or mucosal injury between the two groups.

**Conclusion:**

The results indicate that the TBS-UAS may provide notable advantages over conventional sheaths in RIRS, particularly in improving SFRs and reducing both overall and infectious complication rates. Further large-scale RCTs are needed to confirm these findings and assess long-term outcomes.

## Introduction

Urolithiasis is a prevalent global chronic disorder. In the United States, it impacts around 11% of the population, which translates to roughly one out of nine people being affected.^[1–3]^ The management of renal stones has evolved significantly over the past few decades, with advancements in minimally invasive techniques improving patient outcomes and reducing recovery times^[4,5]^. Retrograde intrarenal surgery (RIRS) has emerged as a preferred approach for the treatment of renal calculi, particularly for stones located in complex anatomical regions or those that are larger than 2 cm[6]. Central to the success of RIRS is the use of ureteral access sheaths (UASs), which facilitate the passage of endoscopic instruments and improve irrigation during the procedure.

**HIGHLIGHTS**

- This meta-analysis was the first to evaluate the comparative effectiveness and safety profiles between TBS-UAS and traditional UAS in RIRS.
- The results indicate that TBS-UAS may provide notable advantages over conventional sheaths in RIRS, particularly in improving SFRs and reducing both overall and infectious complication rates.
- Further large-scale RCTs are needed to confirm these findings and assess long-term outcomes.

Traditional UASs, while effective, present certain limitations, including the risk of ureteral trauma, increased intrarenal pressure, and potential complications related to prolonged use[7]. Recently, the introduction of tip-bendable suction ureteral access sheaths (TBS-UAS) has garnered attention for their potential advantages. These innovative devices allow for enhanced maneuverability within the renal pelvis and calyces, enabling better access to stones in challenging locations and facilitating continuous suction to maintain a clear surgical field.

However, several studies have reported on the efficacy and safety of TBS-UAS compared to traditional sheaths. Despite these promising findings, a comprehensive comparison of TBS-UAS versus traditional sheaths in the context of RIRS remains limited. This systematic review and meta-analysis aims to synthesize the current literature, evaluating the comparative effectiveness and safety of these two types of access sheaths. Unlike prior reviews of suction UAS, this meta-analysis focuses on tip-bendable designs, quantifies their impact on immediate vs. delayed SFR, and resolves safety debates. By analyzing available data, we hope to provide clearer insights into their respective roles in optimizing outcomes for patients undergoing RIRS. In alignment with best practices for transparent scientific reporting, our study adheres to the TITAN 2025 guideline for rigorous methodology disclosure[8].

## Materials and methods

Following PRISMA (Preferred Reporting Items for Systematic Reviews and Meta-Analyses) and AMSTAR (Assessing the Methodological Quality of Systematic Reviews) guidelines, we performed a meta-analysis and systematic review of original studies^[9,10]^. This study rigorously followed the TITAN 2025 reporting guideline[8]; the completed checklist is provided in Supplementary Digital Content File 1, available at: http://links.lww.com/JS9/E771. Consequently, ethical approval and patient consent were not required.

### Literature search

The MEDLINE (via PubMed), Embase (via Ovid), and Cochrane Library databases were systematically searched for eligible studies published from their inception until January 2025. Studies that investigated the efficacy and safety of TBS-UAS in comparison to traditional UAS within the context of retrograde intrarenal stone surgery (RIRS) were included. No language, publication type, or geographic restrictions were applied. The search strategy employed a combination of medical subject headings (MeSH) and non-MeSH terms, specifically (“tip-bendable” OR “tip-flexible”) AND (“suction” OR “aspiration”) AND (“ureteral access sheath”). Additionally, we conducted a manual search of reference lists from prior reviews and pertinent articles to identify further relevant reports. Any discrepancies encountered were resolved through consensus among the co-investigators.

### Inclusion and exclusion criteria

The initial screening of article titles and abstracts was conducted independently by two researchers to identify and exclude ineligible reports, with detailed documentation of exclusion rationales. Full-text evaluations were subsequently performed on studies deemed potentially eligible. Following the selection process, the pertinence of each study was verified. Inclusion criteria were established as follows: (1) inclusion of original research comparing the efficacy and safety of TBS-UAS with traditional UAS in RIRS procedures; (2) studies providing adequate raw data; and (3) original articles presenting prospective or retrospective comparative analyses. Key outcomes included stone-free rates (SFRs), operative time, duration of postoperative hospitalization, and incidence of complications. In instances of duplicate publications, preference was given to the most recent or methodologically superior study. Exclusions were applied to case reports, correspondence, review articles, and conference abstracts. Any discrepancies in study selection were resolved through consensus among the research team.

### Data extraction and methodological quality assessment

Two researchers independently conducted data extraction from the selected studies, with any inconsistencies being resolved through consensus among the research team. A standardized Excel spreadsheet (Microsoft Corporation) was used to record key study characteristics, including primary author, year of publication, research design, participant numbers, stone dimensions, computed tomography (CT) values of stones, anatomical location of stones, operative duration, length of postoperative hospitalization, immediate and final SFRs, and incidence of complications. In cases where essential data were incomplete, corresponding authors were contacted to obtain the necessary information.

Study quality evaluation was performed independently by two researchers using the Newcastle–Ottawa Scale (NOS) version[11], comprising 10 assessment criteria. Each criterion was scored as either “1” (yes) or “0” (no/unclear) based on the extracted study information. The cumulative scores, ranging from 0 to 10 points, were classified into three quality tiers: high quality (8–10 points), moderate quality (5–7 points), and low quality (≤5 points). Evidence certainty was appraised using the GRADE framework (Grading of Recommendations, Assessment, Development, and Evaluations)[12]. Outcomes derived from randomized controlled trials (RCTs) commenced as high-quality evidence; those from observational studies as low quality. Downgrading occurred for risk of bias, inconsistency (*I*^2^ > 50%), or imprecision [confidence intervals (CIs) crossing unity]; upgrading was considered for large effect sizes [relative risk (RR) < 0.5]. Any discrepancies in quality assessment were resolved through collaborative discussion among the research team members.

### Data synthesis and analysis

To assess the comparative efficacy and safety profiles of TBS-UAS relative to conventional UAS in RIRS procedures, statistical analyses were conducted utilizing STATA software (StataCorp Wyb, version 15.0; serial number: 10699393). Comprehensive risk assessments were graphically represented through forest plots, delineating both pooled outcomes and inter-study variability. Heterogeneity was quantified using the *I*^2^ statistic, with predefined thresholds categorizing heterogeneity levels as follows: 0% (none), 25% (minimal), 50% (moderate), and 75% (substantial). Consistent with Cochrane review guidelines, a random-effects model was applied for analyses exhibiting *I*^2^ values exceeding 50%, whereas a fixed-effects model was utilized for analyses with *I*^2^ values below this threshold. A significance level of *P* < 0.05 was adopted for all statistical analyses. Methodological rigor was ensured through stratified subgroup analyses, which were conducted based on study design parameters to examine potential confounding factors associated with methodological approaches and patient demographic characteristics. However, subgroup analyses for stone size/location were unfeasible due to data limitations. The robustness of the findings was assessed through sensitivity analyses, in which each study was systematically excluded to evaluate the consistency of the results. To identify potential sources of heterogeneity, meta-regression analyses were performed employing the restricted maximum likelihood estimation method. The assessment of publication bias was limited by insufficient data availability, thereby precluding the application of Egger’s test[13].

## Results

### Study identification and selection

**Figure 1.**
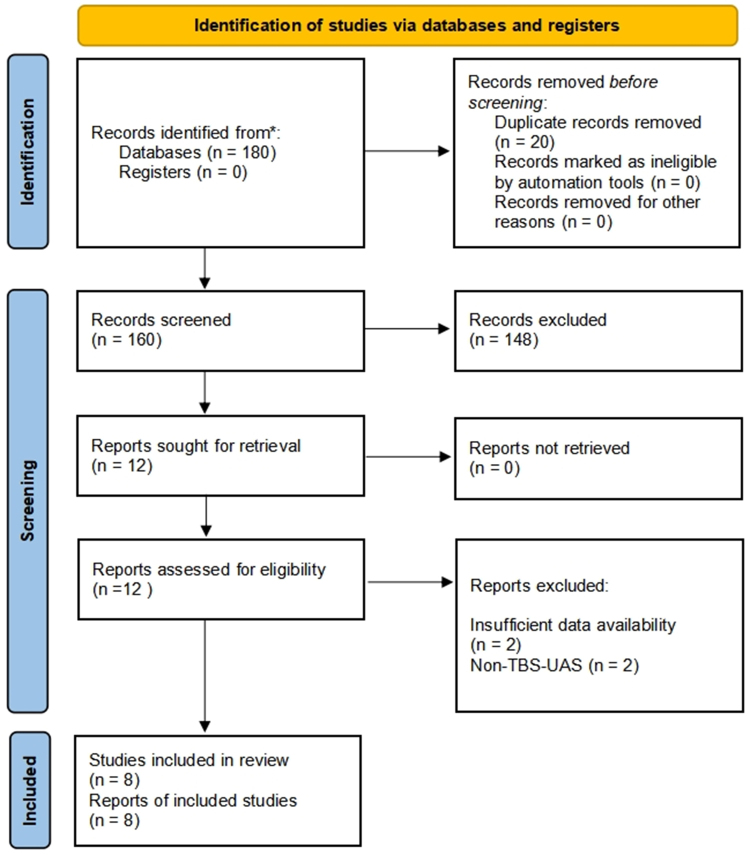
Flow diagram of literature searches according to the Preferred Reporting Items for Systematic Reviews and Meta-Analyses statement.

A total of 180 records were initially identified through our comprehensive search strategy. Following the removal of duplicate entries, 160 studies remained for further evaluation. Subsequent screening of titles and abstracts led to the exclusion of 148 studies, while 12 articles underwent full-text assessment. During the final selection phase, four articles were excluded based on predefined criteria: one due to insufficient data availability and three because they involved non-TBS-UAS (as illustrated in Fig. 1). The systematic review and meta-analysis ultimately included eight original studies,^[14–21]^ encompassing a total of 1981 patients, all of which met the established eligibility criteria.

### Study characteristics

Table 1 presents the fundamental characteristics of the studies included in this analysis. The research comprised eight comparative investigations, with four retrospective studies^[14,15,18,20]^ and four RCTs^[16,17,19,21]^, all published during the period from 2023 to 2025. The participant numbers varied across studies, ranging from 88 to 371 patients. The majority of the studies included patients with a variety of stone sizes, and stone locations, including renal pelvis, upper, middle, and lower calyces. A 200 μm holmium laser fiber was utilized for lithotripsy procedures, with energy settings between 0.5 and 1.0 J and frequency parameters of 20–30 Hz. Non-contrast computerized tomography served as the primary diagnostic tool for evaluating stone-free status post-RIRS, with stone-free criteria defined as either complete absence of fragments or residual fragments measuring less than 2–3 mm. The studies were exclusively published in English-language journals. Further details regarding stone characteristics, including size, CT values, anatomical location, and operative duration, are comprehensively documented in Table 1.

**Table 1:** Characteristics of the included studies

Study	Study design (duration)	Sample size	Age (±SD)	BMI (±SD)	Stone size cm (±SD)	CT value of stone (HU)	Stone location (control)	Stone location (experimental)	Energy used for lithotripsy	SFR definition	Surgical time (min)
Chen H et al. 2024^14^	Retrospective (2019–2023)	Control: 113	Control: 46.35±14.88	Control: 25.66±4.35	Control: 2.68±1.42	NA	Renal pelvis: 0	Renal pelvis: 0	Ho:YAG lithotripsy (1.0 J, 30 Hz), 200 μm fibre	Fragments ≤2 mm (CT scans)	Control: 86.23±20.35
		Experimental: 125									
							Upper calyx: 31	Upper calyx: 43			
								Middle calyx: 67			
											Experimental: 101.17±25.64
			Experimental: 45.62±12.93	Experimental: 25.21±3.77	Experimental: 2.81±1.56		Middle calyx: 69				
							Lower calyx: 13	Lower calyx: 15			
Hu H et al. 2025^15^	Retrospective (2022–2023)	Control: 127	Control: 54 (47–60)	NA	Control: 1.6 (1.4–1.9)	Control: 718 (554–881)	NA	NA	Ho:YAG lithotripsy (0.8 J, 20 Hz), 200 μm fibre	Fragments ≤2 mm (CT scans)	Control: 74 (56–94)
											Experimental: 48 (40–55)
		Experimental: 78									
			Experimental: 55 (38–61)		Experimental: 1.6 (1.3–1.8)	Experimental: 732 (553–923)					
Huang J et al. 2023^16^	RCT (2022–2023)	Control: 257	Control: 56.3±11.6	Control: 26.4±5.1	Control: 1.6±0.5	NA	NA	NA	Ho:YAG lithotripsy (1.2 J, 20 Hz), 200 μm fibre	Fragments ≤3 mm (CT scans)	Control: 40.3±18.9
		Experimental: 114									
			Experimental: 54.5±11.0	Experimental: 26.3±4.2	Experimental: 1.7±0.6						
											Experimental: 37.7±20.1
Uslu M et al. 2024^17^	RCT (2023–2024)	Control: 45	Control: 55 (42–61)	Control: 26 (22–29)	Control: 1.1 (0.8–1.3)	Control: 884 (642–1017)	NA	NA	Ho:YAG lithotripsy (0.5 J, 20 Hz), 273 μm fibre	Fragments ≤3 mm (CT scans)	Control: 62 (59–72)
		Experimental: 43									
			Experimental: 56 (39–69)	Experimental: 27 (23–32)	Experimental: 1.0 (0.8–1.7)	Experimental: 856 (589–1017)					Experimental: 55 (48–65)
Ying Z et al. 2024^18^	Retrospective (2023)	Control: 138	Control: 53.92±14.75	Control: 24.91±3.67	Control: 1.56±0.64	NA	Renal pelvis: 89	Renal pelvis: 67	Ho:YAG lithotripsy (0.8 J, 20 Hz), 200 μm fibre	Fragments ≤2 mm (CT scans)	Control: 65.93±24.99
		Experimental: 103					Upper calyx: 8				
								Upper calyx: 4			
							Middle calyx: 14	Middle calyx: 5			
											Experimental: 69.39±21.69
			Experimental: 53.08±12.99	Experimental: 24.56±3.37	Experimental: 1.55±0.58						
							Lower calyx: 17				
								Lower calyx: 20			
							Multiple calyxes: 10				
								Multiple calyxes: 7			
Yu Y et al. 2024^19^	RCT (2022)	Control: 152	Control: 50.5±11.8	Control: 23.8±2.8	Control: 1.52±0.19	Control: 1303.3±258.2	Renal pelvis: 18	Renal pelvis: 27	Ho:YAG lithotripsy (1.0 J, 15 Hz), 200 μm fibre	Fragments ≤2 mm (CT scans)	Control: 59.9±16.2
								Upper calyx: 25			
		Experimental: 152					Upper calyx: 24				
								Middle calyx: 36			
							Middle calyx: 33				
			Experimental: 51.1±12.2	Experimental: 24.3±2.8	Experimental: 1.55±0.2	Experimental: 1325.1±262.2					
							Lower calyx: 59				
											Experimental: 56.5±13.9
								Lower calyx: 48			
							Multiple calyxes: 18				
								Multiple calyxes: 16			
Zhang Z et al. 2023^20^	Retrospective (2021–2022)	Control: 112	Control: 46.75±11.87	Control: 23.54±3.37	Control: 1.82±0.45	NA	Renal pelvis: 43	Renal pelvis: 41	Ho:YAG lithotripsy (0.6 J, 20 Hz), 200 μm fibre	Fragments ≤2 mm (CT scans)	Control: 59.36±15.59
		Experimental: 102						Upper calyx: 10			
							Upper calyx: 9				
											Experimental: 55.25±11.42
								Middle calyx: 9			
							Middle calyx: 11				
			Experimental: 47.69±9.18	Experimental: 24.25±2.97	Experimental: 1.85±0.47						
							Lower calyx: 15	Lower calyx: 12			
							Multiple calyxes: 34	Multiple calyxes: 30			
Zhu W et al. 2024 ^21^	RCT (2023–2024)	Control: 160	Control: 52 (40–61.8)	Control: 24.2 (21.9–27)	Control: 1.1 (0.9–1.6)	Control: 897.5 (707.8–1155.8)	Renal pelvis: 11	Renal pelvis: 18 Upper calyx: 22	Ho:YAG lithotripsy (0.8 J, 20 Hz), 200 μm fibre	Fragments ≤2 mm (CT scans)	Control: 40.9±19.1
		Experimental: 160									
							Uppercalyx: 52				
								Middle calyx: 13			
					Experimental: 1.4 (1.0–2.0)	Experimental: 932 (682.5–1145)	Middle calyx: 21				
							Lower calyx: 59	Lower calyx: 52			
											Experimental: 45±23.2
								Multiple calyxes: 73			
			Experimental: 53 (45–64)	Experimental: 24.8 (22.4–26.5)							
							Multiple calyxes: 62				

BMI, body mass index; CT, computed tomography; SD, standard deviation; NA, not available; SFR, stone-free rate.

### Methodological quality assessment

The methodological quality of the included studies was rigorously assessed utilizing the NOS criteria. Of the studies analyzed, seven investigations^[14,16–21]^ attained scores between 9 and 10 points, thereby qualifying as high-quality research. In contrast, one study was assigned an 8-point score, categorizing it as moderate in quality[15].

The GRADE assessment reveals variable certainty across evaluated outcomes. For immediate SFRs, the evidence quality is moderate, supported by consistent results but limited by methodological shortcomings in included studies. Final SFR data similarly achieves moderate confidence, though with slightly diminished effect size. Surgical duration and postoperative hospitalization outcomes both demonstrate low-quality evidence, primarily due to imprecise effect estimates and study design limitations. Across all endpoints, risk of bias constitutes the most significant constraint, stemming largely from inadequate blinding procedures and allocation concealment in source studies (Table 3).

### Immediate and final SFR

**Figure 2.**
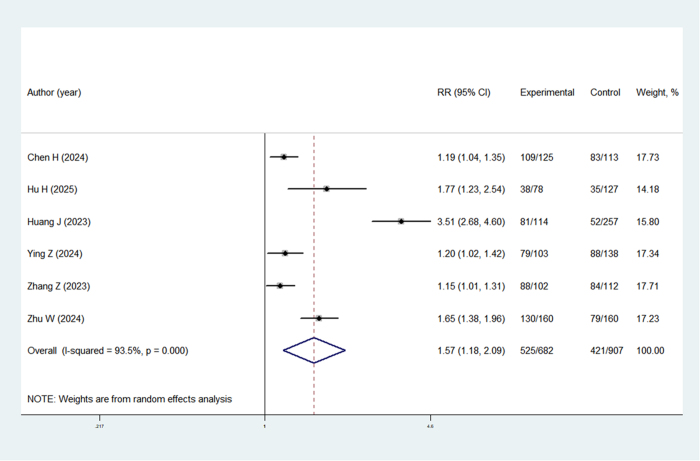
Immediate SFR of TBS-UAS compared to traditional UAS.

**Figure 3.**
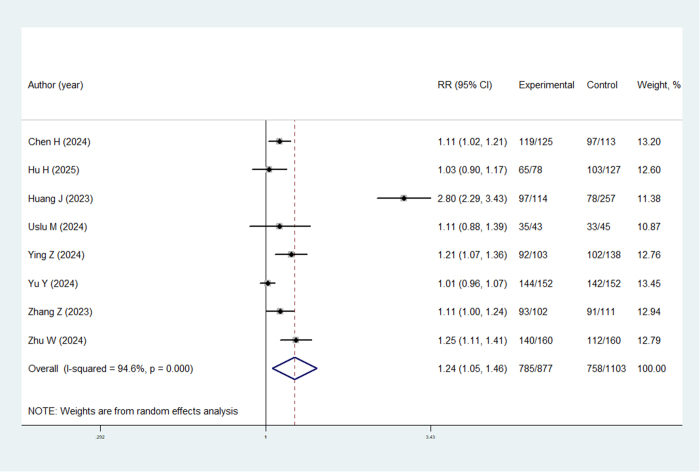
Final SFR of TBS-UAS compared to traditional UAS.

A significant disparity in immediate SFR was demonstrated between TBS-UAS and traditional UAS groups through a pooled analysis of six investigations (RR = 1.57, 95% CI: 1.18–2.09; *P* = 0.002) (Fig. 2). Furthermore, the TBS-UAS cohort exhibited markedly higher final SFR values at the 1-month post-RIRS assessment when contrasted with the traditional UAS group (RR = 1.24, 95% CI: 1.05–1.46; *P* = 0.011) (Fig. 3).

**Figure 4.**
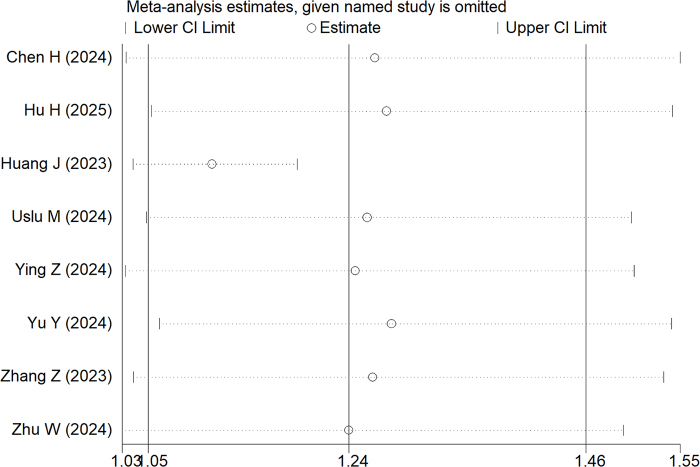
Results of sensitivity analyses.

The research incorporated subgroup evaluations stratified by varying methodological approaches. While retrospective investigations consistently demonstrated enhanced final SFR outcomes for TBS-UAS (RR = 1.11, 95% CI: 1.05–1.18; *P* < 0.05), RCTs failed to replicate this statistical significance (RR = 1.40, 95% CI: 0.88–2.23; *P* = 0.150). Sensitivity assessments confirmed the stability of these findings, as the exclusion of individual studies did not substantially modify the overall conclusions regarding final SFR, thereby substantiating the robustness of the outcomes (Fig. 4).

To examine the influence of potential confounding variables, including study methodology, meta-regression analyses were implemented. The resulting adjusted *P*-values of 0.37 suggested minimal explanatory contribution from these regressors to the response variables. This comprehensive analytical approach reinforced the validity and reliability of the study’s principal findings.

### Surgical time and postoperative hospital stay

No statistically significant variation in surgical duration was observed when comparing the TBS-UAS cohort with the traditional UAS group (MD = 0.05, 95% CI: −0.21–0.32; *P* = 0.701). In parallel, the length of hospital stay following surgery demonstrated comparable outcomes across both treatment arms, with no significant disparity detected (MD = −0.10, 95% CI: −0.21–0.01; *P* = 0.074) (Table 2).

**Table 2:** Results of meta-analyses on secondary outcomes

Outcomes	Studies, *N*	MD (95% CI)	*P*-value	*I*^2^ (%)
Surgical time	6	0.05 (−0.21–0.32)	0.701	86.4
Postoperative hospital stay	5	−0.10 (−0.21–0.01)	0.704	0
**Outcomes**	**Studies, *N***	**RR (95% CI)**	***P*-value**	***I*^2^ (%)**
Complication rates	5	0.41 (0.29–0.56)	<0.05	0
Fever	8	0.37 (0.25–0.53)	<0.05	0
Intraoperative bleeding	2	0.32 (0.03–3.03)	0.318	0
Mucosal injury	3	0.50 (0.22–1.18)	0.113	0.2

MD, mean difference; RR, relative risk.

**Table 3:** Summary of findings from the GRADE evidence assessment

Quality assessment							No of patients		Effect		Quality	Importance
No of studies	Design	Risk of bias	Inconsistency	Indirectness	Imprecision	Other considerations	TBS-UAS	UAS	Relative (95% CI)	Absolute		
**Immediate postoperative SFR**												
6	RCTs and retrospective studies	Very Serious^a^	Not serious	Not serious	Not serious	None	525/682	421/907	RR: 1.57 (1.18– 2.09)	439 more per 1000 (139–229 more)	⊕⊕⊕○	
**Final postoperative SFR**												
8	RCTs and retrospective studies	Very serious^a^	Not serious	Not serious	Not serious	None	785/877	785/1103	RR: 1.24 (1.05–1.46)	185 more per 1000 (123–254 more)	⊕⊕⊕○	
**Surgical time**												
6	RCTs and retrospective studies	Very serious^a^	Not serious	Not serious	Not serious	None	756	932	MD: 0.05 (−0.21 to 0.32)	0.05 min longer (−0.21 to 0.32)	⊕⊕○○	
**Postoperative hospital stay**												
5	RCTs and retrospective studies	Very serious^a^	Not serious	Not serious	Not serious	None	642	675	MD: −0.10 (−0.21 to 0.01)	0.10 days shorter (−0.21 to 0.01)	⊕⊕○○	

^a^ All of the trials did not report concrete details on allocation concealment and the blinding of participants and outcome assessors.

### Complication rates

A significant reduction in the overall complication rate was observed with the use of TBS-UAS (RR = 0.41, 95% CI: 0.29–0.56; *P* < 0.05), along with a decreased occurrence of fever (RR = 0.37, 95% CI: 0.25–0.53; *P* < 0.05). The analysis of specific complications, such as intraoperative bleeding (RR = 0.32, 95% CI: 0.03–3.03; *P* = 0.318) and mucosal injury (RR = 0.50, 95% CI: 0.22–1.18; *P* = 0.113), revealed no statistically significant differences between the groups (Table 2).

## Discussion

### Main findings

A systematic review and meta-analysis was performed to evaluate the comparative effectiveness and safety profiles between TBS-UAS and traditional UAS in RIRS. The findings demonstrate that TBS-UAS potentially offers significant clinical benefits over standard sheaths during RIRS procedures, specifically in enhancing SFRs while decreasing the incidence of both general and infection-related complications. Through sensitivity analysis, the robustness of these outcomes remained consistent upon sequential exclusion of individual studies, yet meta-regression analysis failed to determine specific variables contributing to the observed inter-study heterogeneity.

### Implications for clinical practice

Although RIRS has gained widespread clinical application, the persistence of residual stone fragments postoperatively continues to pose significant clinical challenges. Radiological assessments using CT scans have demonstrated that approximately 38% of renal units exhibit residual fragments exceeding 2 mm following RIRS procedures^[22,23]^. Clinical evidence suggests a nine-fold increase in the likelihood of requiring secondary surgical interventions among patients with residual fragments >2 mm compared to those with fragments ≤2 mm^[24,25]^. These fragments frequently cause complications such as renal colic and hematuria, emphasizing the critical need for achieving complete stone clearance during the initial surgical intervention.

The implementation of TBS-UAS technology has demonstrated enhanced fragment clearance through its direct suction mechanism, effectively maintaining intraoperative visualization while minimizing residual fragments and ensuring comprehensive stone fragmentation. In a comparative retrospective analysis conducted by Zhu *et al*, suctioning UAS demonstrated superior immediate SFRs (82.4%) compared to conventional UAS (71.5%) on postoperative day 1 (*P* = 0.02), although this difference diminished at the 1-month follow-up (88.8% vs. 82.9%; *P* = 0.13)[26]. Notably, Zeng *et al* documented an exceptional immediate SFR of 97.3% in their cohort of 74 patients undergoing modified suctioning UAS procedures, surpassing our findings. However, it should be noted that their study population predominantly consisted of ureteral stone cases (*n* = 67) with only a limited number of flexible ureteroscopic procedures (*n* = 7)[27].

The incidence of urinary tract infections following RIRS procedures ranges between 1.7% and 18.8%, representing one of the most prevalent complications[28]. Our investigation revealed that TBS-UAS implementation was associated with a significant reduction in postoperative febrile episodes, potentially indicating its protective role against infectious complications. Unlike conventional UAS devices positioned at the ureteropelvic junction, the TBS-UAS system offers adjustable positioning within the renal pelvis, enabling access to specific calyces while maintaining optimal irrigation and suction capabilities. This design feature prevents ureteropelvic junction mucosal obstruction of the UAS opening and facilitates maintenance of low intrarenal pressures.

Our study findings diverge from previous reports regarding operative duration, demonstrating comparable surgical times between the two groups. While Ding *et al* reported superior stone removal efficacy with suction UAS compared to traditional systems[29], our experience indicates that the TBS-UAS suction process still requires considerable time investment. Although basket retrieval methods may offer time efficiency advantages, they present technical challenges in complete removal of minute fragments. Furthermore, our data revealed no significant disparity in ureteral wall injury rates between groups. The TBS-UAS system’s soft, collapsible tip design facilitates smoother insertion with reduced resistance, suggesting potential benefits in minimizing ureteral trauma that warrant further investigation.

The operational advantages of TBS-UAS can be attributed to several factors. First, the 270° tip-flexible design enables precise alignment with anatomically challenging sites, particularly steep lower-pole infundibulopelvic angles, permitting direct stone targeting under fluoroscopy. This maneuverability facilitates specialized techniques such as dynamic sheath rotation during lithotripsy to clear dependent calyceal fragments. Second, integrated suction maintains low-pressure irrigation, continuously evacuating debris to prevent retropulsion and reduce residual fragments. The resultant unobstructed visual field not only optimizes laser fragmentation efficiency but also mitigates post-operative hydronephrosis and fornix rupture risks associated with traditional UAS.

Nevertheless, it should be noted that the use of TBS-UAS also has its limitations. The cost of the device is relatively higher, which may limit its widespread application in some healthcare settings. Moreover, the learning curve for surgeons to become proficient in using it could pose a challenge initially. Future research should focus on optimizing the design to further reduce costs and conducting more training programs to shorten the learning curve[30].

In conclusion, our systematic review and meta-analysis, along with the insights from recent literature, suggest that the TBS-UAS holds significant advantages over the traditional UAS in RIRS in terms of SFR and infectious complication reduction. Based on our findings, we recommend: (1) TBS-UAS for stones in difficult locations (e.g., lower pole stone); (2) its integration into sepsis-reduction protocols; and (3) cost-effectiveness analyses comparing initial investment against savings from reduced reoperations. With appropriate surgical techniques and further improvements, it has the potential to become the standard of care in the future, benefiting more patients undergoing this type of urological procedure.

### Strengths and limitations

The meta-analysis exhibited several crucial strengths. Generally, this meta-analysis was the first to evaluate the comparative effectiveness and safety profiles between TBS-UAS and traditional UAS in RIRS, and subgroup analyses were conducted by different study designs to determine whether these variables influenced the level of heterogeneity according to the PRISMA guidelines. Importantly, the results of the meta-regression and sensitivity analyses validated the reliability and rationality of our study.

Despite our efforts, this study has certain limitations. One of the main challenges was the heterogeneity among the included studies. Although we attempted to minimize it through strict selection criteria, differences in patient characteristics, surgical techniques, and outcome definitions still existed. For example, the stone burden and location varied across studies, which could potentially influence the performance of the access sheaths. These sources of heterogeneity may have affected the precision and generalizability of our pooled estimates. Despite employing random-effects models, sensitivity analyses, and meta-regression, the sources of high heterogeneity in operative time remained statistically unexplained. Clinical factors, including surgeon experience, stone complexity, and unmeasured anatomical variations, likely contributed to this variability. Our inability to perform subgroup analyses for stone size, location, or laser parameters due to insufficient primary data underscores the need for future studies to report stratified outcomes. Another limitation pertains to the quality of the included studies. Some of the original studies had relatively small sample sizes, which might limit the statistical robustness of their individual findings. Additionally, the risk of bias assessment indicated that certain studies had methodological flaws, such as incomplete blinding or lack of proper randomization. These issues could have introduced biases into our meta-analysis and affected the overall conclusions. Furthermore, the follow-up duration in some studies was relatively short. Long-term outcomes, such as potential late complications or the durability of the effects observed, might not have been fully captured. This shortcoming restricts our ability to make definitive statements about the long-term superiority or equivalence of the two types of sheaths. Moreover, the search was limited to English-language literature, which may have excluded relevant studies published in other languages. Although English is the dominant language in scientific publishing, there is a possibility that valuable data from non-English sources could have influenced our results. Finally, the limited number of included articles may lead to less comprehensive results. Hence, we anticipate future high-quality multicenter studies will validate our findings and refine patient selection criteria. Such advancements are essential to optimize clinical protocols and ultimately enhance stone-free outcomes for complex patient populations.

## Conclusion

In conclusion, this meta-analysis comparing the TBS-UAS with the traditional UAS in RIRS has yielded significant insights. The TBS-UAS demonstrated clear advantages in enhancing surgical efficiency, notably in facilitating stone removal. It also showed promising trends in improving safety outcomes by reducing certain postoperative complications. Looking ahead, these findings pave the way for future research. Clinicians should consider incorporating the TBS-UAS where appropriate, while researchers need to conduct further large-scale, well-designed studies. Long-term follow-up and multicenter trials are warranted to solidify the evidence base, ultimately aiming to optimize patient care and surgical practices in urology. This will ensure the continued evolution and refinement of techniques in retrograde intrarenal stone surgery.

## Data Availability

Data have been listed below each figure and table.

## Research registration unique identifying number (UIN)

Name of the registry: PROSPERO database. Unique identifying number or registration ID: CRD42025637043.

## References

[1] Scales Jr CD Smith AC Hanley JM Saigal CS. Urologic Diseases in America Project. Prevalence of kidney stones in the United States. Eur Urol 2012;62:160–65. 22498635 10.1016/j.eururo.2012.03.052 PMC3362665

[2] Hill AJ Basourakos SP Lewicki P. Incidence of kidney stones in the United States: the continuous national health and nutrition examination survey. J Urol 2022;207:851–56. 34854755 10.1097/JU.0000000000002331

[3] Zeng G Mai Z Xia S. Prevalence of kidney stones in China: an ultrasonography based cross-sectional study. BJU Int 2017;120:109–16. 28236332 10.1111/bju.13828

[4] Desai M Sun Y Buchholz N. Treatment selection for urolithiasis: percutaneous nephrolithomy, ureteroscopy, shock wave lithotripsy, and active monitoring. World J Urol 2017;35:1395–99. 28303335 10.1007/s00345-017-2030-8

[5] Geraghty RM Jones P Somani BK. Worldwide trends of urinary stone disease treatment over the last two decades: a systematic review. J Endourol 2017;31:547–56. 28095709 10.1089/end.2016.0895

[6] Wu W Yang Z Xu C. External physical vibration lithecbole promotes the clearance of upper urinary stones after retrograde intrarenal surgery: a prospective, multicenter, randomized controlled trial. J Urol 2017;197:1289–95. 28063841 10.1016/j.juro.2017.01.001

[7] Traxer O Thomas A. Prospective evaluation and classification of ureteral wall injuries resulting from insertion of a ureteral access sheath during retrograde intrarenal surgery. J Urol 2013;189:580–84. 22982421 10.1016/j.juro.2012.08.197

[8] Agha RA Mathew G Rashid R. Transparency in the reporting of Artificial INtelligence – the TITAN guideline. Prem J Sci 2025;10:100082.

[9] Page MJ McKenzie JE Bossuyt PM. The PRISMA 2020 statement: an updated guideline for reporting systematic reviews. Int J Surg 2021;88:105906. 33789826 10.1016/j.ijsu.2021.105906

[10] Shea BJ Reeves BC Wells G. AMSTAR 2: a critical appraisal tool for systematic reviews that include randomised or non-randomised studies of healthcare interventions, or both. BMJ 2017;358:j4008. 28935701 10.1136/bmj.j4008 PMC5833365

[11] Wells GA Shea B O’Connell D. The Newcastle-Ottawa Scale (NOS) for Assessing the Quality of Nonrandomized Studies in Meta-analysis. Ottawa, ON, Canada: Ottawa Hospital Research Institute; 2014. http://www.ohri.ca/programs/clinical_epidemiology/oxford.asp

[12] GRADE Working Group, Guyatt GH Oxman AD Vist GE. GRADE: an emerging consensus on rating quality of evidence and strength of recommendations. BMJ 2008;336:924–26. 18436948 10.1136/bmj.39489.470347.AD PMC2335261

[13] Egger M Davey Smith G Schneider M Minder C. Bias in meta-analysis detected by a simple, graphical test. BMJ 1997;315:629–34. 9310563 10.1136/bmj.315.7109.629 PMC2127453

[14] Chen H Xiao J Ge J Liu T. Clinical efficacy analysis of tip-flexible suctioning ureteral access sheath combined with disposable flexible ureteroscope to treat 2-4 cm renal stones. Int Urol Nephrol 2024;56:3193–99. 38717576 10.1007/s11255-024-04072-y PMC11405463

[15] Hu H Qin M Yang L. Analysis of the effectiveness and safety of 7.5 Fr ultra-thin flexible ureteroscope combined with a tip-flexible suctioning ureteral access sheath for the treatment of kidney stones. Int Urol Nephrol 2025;57:817–23. 39521733 10.1007/s11255-024-04269-1 PMC11821792

[16] Huang J Yang Y Xie H. Vacuum-assisted dedusting lithotripsy in the treatment of kidney and proximal ureteral stones less than 3 cm in size. World J Urol 2023;41:3097–103. 37698634 10.1007/s00345-023-04595-6

[17] Uslu M Yildirim Ü Ezer M. Comparison of tip-bendable aspiration-assisted and standard access sheaths in the treatment of lower calyceal stones. Rev Assoc Med Bras (1992) 2024;70:e20241033. 39699482 10.1590/1806-9282.20241033 PMC11656536

[18] Ying Z Dong H Li C. Efficacy analysis of tip-flexible suction access sheath during flexible ureteroscopic lithotripsy for unilateral upper urinary tract calculi. World J Urol 2024;42:626. 39499350 10.1007/s00345-024-05325-2

[19] Yu Y Chen Y Zhou X. Comparison of novel flexible and traditional ureteral access sheath in retrograde intrarenal surgery. World J Urol 2024;42:7. 38175210 10.1007/s00345-023-04697-1 PMC10766707

[20] Zhang Z Xie T Li F. Comparison of traditional and novel tip-flexible suctioning ureteral access sheath combined with flexible ureteroscope to treat unilateral renal calculi. World J Urol 2023;41:3619–27. 37821778 10.1007/s00345-023-04648-w PMC10693513

[21] Zhu W Liu S Cao J. Tip bendable suction ureteral access sheath versus traditional sheath in retrograde intrarenal stone surgery: an international multicentre, randomized, parallel group, superiority study. EClinicalMedicine 2024;74:102724. 39070176 10.1016/j.eclinm.2024.102724 PMC11277316

[22] Danilovic A Cavalanti A Rocha BA. Assessment of residual stone fragments after retrograde intrarenal surgery. J Endourol 2018;32:1108–13. 30398369 10.1089/end.2018.0529

[23] Rippel CA Nikkel L Lin YK. Residual fragments following ureteroscopic lithotripsy: incidence and predictors on postoperative computerized tomography. J Urol 2012;188:2246–51. 23083650 10.1016/j.juro.2012.08.040

[24] Raman JD Bagrodia A Gupta A. Natural history of residual fragments following percutaneous nephrostolithotomy. J Urol 2009;181:1163–68. 19152935 10.1016/j.juro.2008.10.162

[25] Chew BH Brotherhood HL Sur RL. Natural history, complications and re-intervention rates of asymptomatic residual stone fragments after ureteroscopy: a report from the EDGE research consortium. J Urol 2016;195:982–86. 26585680 10.1016/j.juro.2015.11.009

[26] Zhu Z Cui Y Zeng F Li Y Chen Z Hequn C. Comparison of suctioning and traditional ureteral access sheath during flexible ureteroscopy in the treatment of renal stones. World J Urol 2019;37:921–29. 30120500 10.1007/s00345-018-2455-8

[27] Zeng G Wang D Zhang T Wan SP. Modified access sheath for continuous flow ureteroscopic lithotripsy: a preliminary report of a novel concept and technique. J Endourol 2016;30:992–96. 27443243 10.1089/end.2016.0411

[28] Zhang H Jiang T Gao R. Risk factors of infectious complications after retrograde intrarenal surgery: a retrospective clinical analysis. J Int Med Res 2020;48:300060520956833. 32993406 10.1177/0300060520956833 PMC7536499

[29] Ding J Su T Zhang X. Omnidirectional (Flexible) ureteral access sheath: safety, efficacy, and initial experience report. J Endourol 2023;37:1184–90. 37725564 10.1089/end.2023.0358

[30] Vassileva J Zagorska A Karagiannis A. Radiation exposure of surgical team during endourological procedures: International Atomic Energy Agency-South-Eastern European Group for urolithiasis research study. J Endourol 2021;35:574–82. 32791856 10.1089/end.2020.0630

